# Structures of Two Melanoma-Associated Antigens Suggest Allosteric Regulation of Effector Binding

**DOI:** 10.1371/journal.pone.0148762

**Published:** 2016-02-24

**Authors:** Joseph A. Newman, Christopher D. O. Cooper, Anette K. Roos, Hazel Aitkenhead, Udo C. T. Oppermann, Hearn J. Cho, Roman Osman, Opher Gileadi

**Affiliations:** 1 Structural Genomics Consortium, University of Oxford, ORCRB, Roosevelt Drive, Oxford, OX3 7DQ, United Kingdom; 2 NDORMS, University of Oxford, Botnar Research Centre, Oxford, OX3 7LD, United Kingdom; 3 Tisch Cancer Institute, Mt Sinai School of Medicine, Icahn 15-20B 1425 Madison Avenue, New York, NY, 10029, United States of America; 4 Department of Structural and Chemical Biology, Box 1677, Mount Sinai School of Medicine, New York, NY, 10029, United States of America; Mie University Graduate School of Medicine, JAPAN

## Abstract

The MAGE (melanoma associated antigen) protein family are tumour-associated proteins normally present only in reproductive tissues such as germ cells of the testis. The human genome encodes over 60 MAGE genes of which one class (containing MAGE-A3 and MAGE-A4) are exclusively expressed in tumours, making them an attractive target for the development of targeted and immunotherapeutic cancer treatments. Some MAGE proteins are thought to play an active role in driving cancer, modulating the activity of E3 ubiquitin ligases on targets related to apoptosis. Here we determined the crystal structures of MAGE-A3 and MAGE-A4. Both proteins crystallized with a terminal peptide bound in a deep cleft between two tandem-arranged winged helix domains. MAGE-A3 (but not MAGE-A4), is predominantly dimeric in solution. Comparison of MAGE-A3 and MAGE-A3 with a structure of an effector-bound MAGE-G1 suggests that a major conformational rearrangement is required for binding, and that this conformational plasticity may be targeted by allosteric binders.

## Introduction

MAGE genes were first identified as antigens recognized by cytolytic T lymphocytes from a MZ-2 human melanoma cell line [1], and due to the fact that in many cases their expression is strictly limited to tumour cells, are considered attractive targets for immunotherapeutic treatment of a broad spectrum of cancer types. Subsequent work has established the existence of more than 60 MAGE family proteins in humans which have been further classified based on expression pattern into two classes, class I being expressed exclusively in malignant tumours and male germ cells, whilst class II being expressed ubiquitously in normal adult human cells [2]. Further classification of MAGE genes into subfamilies has been performed based on chromosomal clustering with class I constituting 15 MAGE-A genes, 17 MAGE-B genes and 7 MAGE-C genes all clustered to discrete locations on the X chromosome [3]. The silenced expression of proteins in the class I MAGE family genes in normal adult cells is thought to be achieved in some cases by CpG methylation of Ets consensus sites within the promoters [4], and this silencing was found to be reversed in a broad spectrum of tumours due to DNA hypomethylation [5]. All MAGE family members contain a shared core MAGE homology domain (MHD) which is a ~ 200 amino acid long domain, which is well conserved across the entire MAGE family (individual human MHD’s share more than 40% sequence identity). In addition to the MHD both N-terminal and C-terminal domains are present, which show little sequence similarity beyond their immediate subfamily neighbours. Evolutionary analysis suggests that class I MAGE proteins are under positive selection pressure and have diversified in the hominin lineage to acquire additional functions [6].

MAGE-A3 and MAGE-A4 are two of the better studied members of the MAGE family due to their promising status as targets for cancer diagnosis, prognosis and immunotherapy. Both genes are strongly expressed in various cancer types, with MAGE-A3 expression observed in 60% of malignant melanomas [7], 45% of non-small-cell lung carcinomas (NSCLC) [8], 70% of Durie-Salmon stage III multiple myelomas [9] and 37% of bladder cancers [10]. MAGE-A3 was more commonly detected in metastatic (76%) vs primary (36%) melanoma lesions [7], and in relapsed (77%) vs newly diagnosed, untreated (36%) multiple myeloma cases [11]. MAGE-A3 expression by immunohistochemistry or gene expression profiling was associated with poorer outcome in NSCLC and multiple myeloma [8, 12]. MAGE-A4 is expressed in 38% of nonmuscle-invasive bladder carcinoma tumours, 48% of muscle invasive tumours, 65% of carcinomas in situ and 73% of lymph node metastases [13]. Although these findings in primary tumours suggest a pathogenic role for class I MAGE in cancer, until recently it was not clear whether they are “drivers” or “passengers” of the tumorigenic process. It is now apparent that class I MAGE are likely to be oncogenes that promote growth and survival of tumour cells. Expression of MAGE-A3 and MAGE-A6 (a protein that is almost identical in sequence (98% identity), chromosomal location and expression to MAGE-A3) was sufficient to drive the transformation of human colonic epithelial cells to anchorage independent growth, and knockdown causing significant reduction in cancer cell survival [14]. MAGE-A3 knockdown in human multiple myeloma cell lines and primary cells resulted in apoptosis, and in this context it appeared to regulate p53-dependent and independent survival mechanisms [11]. The biochemical basis for this activity appears to be exerted through binding to and modulating the activities of RING domain containing E3 ubiquitin ligases [15, 16]. Surprisingly, given the conservation within the MAGE family, the interaction of various family members with specific RING domain containing proteins was not found to involve the conserved RING domain. Rather, each MAGE protein appears to recognize a unique region on its RING partner, suggesting that MAGE proteins are able to function as a versatile and adaptable protein interaction module. MAGE-A3 was found to interact with the nuclear scaffolding protein and E3 ligase TRIM28 (also known as KAP1), via its RING-B box coiled coil (RBCC) motif [15], and this interaction was found to stimulate the E3 ligase activity leading to degradation of various targets with roles in apoptosis such as the tumour suppressor p53[15, 17], the KRAB zinc finger transcription factor ZNF382[18], and the metabolic regulator AMP activated protein kinase (AMPK)[14]. In contrast to MAGE-A3, relatively little is known about the possible interactions of MAGE-A4 with RING containing ligases. Despite sharing 69% sequence identities, there is considerable evidence for a distinct and possibly contrasting roles for MAGE-A4 in apoptosis and tumour cell proliferation, with the C-terminal region of MAGE-A4 has been found to interact with gankyrin [19] and Miz-1 [20], and induce rather than suppress apoptosis.

Early efforts to exploit MAGE proteins in cancer therapy have focussed on immunotherapeutic approaches, culminating in two phase III clinical trials of a recombinant MAGE-A3 protein vaccine in melanoma and NSCLC [21]. The results of these trials were disappointing, as they did not meet their primary endpoints. However, the revelation of class I MAGE proteins as drivers of oncogenesis has opened the exciting potential to target the cellular function of MAGE proteins in cancer therapy. To gain insights into the structural basis for the functional differences between MAGE proteins and to provide a molecular framework for the rational design of novel compounds targeting MAGE protein function, we have determined the crystal structures of the MAGE homology domains of MAGE-A3 and MAGE-A4. Both proteins crystallized in an unusual arrangement with extended terminal peptides sequences occupying a cleft between the two WH domains. Comparisons with other MAGE family members provide insights into the function and utility of the MHD as a protein-protein interaction module and allow us to suggest a possible strategy for MAGE inhibition based on compounds binding preferentially to distinct conformational states. Finally we have re-examined previous structural data of a complex between a MAGE protein and an E3 ligase, the MAGE-G1-NSE-1 complex, and propose a possible alternative arrangement for the complex with distinct topological properties which offer possible new insights into MAGE family regulation and interaction.

## Materials and Methods

### Cloning and protein expression

Plasmid DNA templates for full-length human MAGE-A3 (IMAGE id 3345801) and MAGE-A4 (IMAGE id 4394783) were obtained from the Mammalian Gene Collection (Source BioScience, Nottingham, UK). Regions corresponding to the MHD domains of MAGE-A3 and MAGE-A4 (residues 104–314 and 101–317 respectively), and a C-terminally truncated version of MAGE-A3 (residues 104–294) were amplified by PCR. MAGEA4 fragments were cloned into vector pNIC28-Bsa4, which appends a Tobacco Etch Virus (TEV) protease-cleavable His_6_ tag at the N-terminal. MAGEA3 fragments were cloned into vector pNH-TrxT (GU269914) which appends a TEV-cleavable His_6_-thioredoxin tag, as described elsewhere [22]. For overexpression, plasmids were transformed into BL21 (DE3) Rosetta-R3, and cultures were grown in UltraYield baffled flasks (Thomson Instrument Company, CA, USA) in terrific broth medium containing 50 μg/ml kanamycin, at a temperature of 37°C to an optical density of 2–3, at which point the cultures were cooled to 18°C and expression was induced by addition of 0.3 mM Isopropyl β-D-1-thiogalactopyranoside, cells were harvested 18hr after induction. For production of selenomethionine incorporated MAGE-A4, cells were inoculated into 6x 10-ml of LB medium containing 50 μg/ml kanamycin and 35 μg/ml chloramphenicol and grown overnight at 37°C. Cultures were harvested by centrifugation, washed twice with M9 minimal medium and resuspended in 10 ml of M9 minimal medium. These cultures were used to inoculate 1L of M9 minimal medium, which was grown in 2.5L UltraYield baffled flasks until OD_600_ of 0.80. Selenomethioine was added to 25mg/L along with leucine, isoleucine and valine to 50mg/L and lysine, threonine, and phenylalanine to 100mg/L (all amino acids dissolved in 0.2M HEPES pH 7.5). Cultures were grown for a further 1.5 hours until OD_600_ of 1.2 and then cooled to 18°C for 1 hour. Additional selenomethioine was added (final total concentration of 75mg/L). IPTG was added to 0.1 mM, and growth continued at 18°C overnight.

### Protein Purification

For purification of both MAGE-A3 constructs cell pellets were thawed and resuspended in buffer A (50 mM HEPES pH 7.5, 500 mM NaCl, 5% glycerol, 10 mM imidazole, 0.5 mM TCEP) and disrupted by sonication. Cell debris and nucleic acids were removed by centrifugation at 50000 x g for 1 hour at 4°C. The supernatants were applied to a 3 ml Ni-IDA IMAC gravity flow column, washed with wash buffer (buffer A with 30 mM imidazole), and eluted with 5CV of elution buffer (buffer A with 300mM imidazole). Proteins were incubated overnight at 4°c in the presence of TEV protease (1:50 mass ratio) whilst being dialysed, using 3.5 kDa MWCO Snakeskin membrane (Thermo Fisher Scientific, Rockford, IL, USA) into buffer B (20 mM HEPES, pH 7.5, 500 mM NaCl, 5% glycerol, 0.5 mM TCEP). TEV protease and contaminating proteins were removed by reapplication of dialysed proteins to a Ni-IDA IMAC column (2 ml CV). Proteins passing through the column were pooled and concentrated, using a 10 kDa MWCO centrifugal concentrator, to 1ml before loading onto a HiLoad 16/60 Superdex S75 gel filtration column equilibrated in buffer B. Fractions containing MAGE-A3 were pooled and diluted 10 fold in dH_2_O to 50 mM NaCl and applied to a 1ml Mono Q anion exchange column equilibrated in 50 mM Hepes pH 7.5, 50 mM NaCl. Proteins were eluted with a linear gradient to 50 mM Hepes pH 7.5, 1M NaCl over 30 CV, and fractions containing MAGE-A3 were pooled and concentrated.

MAGE-A4 was purified as for MAGE-A3 but with the omission of the TEV cleavage, Ni-IDA rebind and anion exchange steps. In both cases proteins were identified by SDS PAGE, although it was found that MAGE-A3 and MAGE-A4 showed unusual behaviour on SDS-PAGE, migrating as two bands connected by smearing with the proportion of the upper band being concentration dependant. This was found to be the case regardless of the construct boundaries or presence or absence of a tag. The identity of both bands belonging to MAGE proteins was confirmed by tryptic digestion and MS/MS analysis. Concentrations were determined by measurement at 280 nM (Nanodrop) using the calculated molecular mass and extinction coefficients.

### Crystallization and Structure Determination

For crystallization, MAGE-A3 and MAGE-A4 were concentrated to 5 mg/ml and 9 mg/ml respectively and sitting drop vapour diffusion crystallization trials were set up with a Mosquito (TTP Labtech) crystallisation robot. Crystals of native MAGE-A4 were observed to grow at 4°C from conditions containing 2.6 M NaCl, 0.1 M Tris-HCl pH 8 and were cryoprotected by transfer to a solution containing 20% (w/v) D-glucose prior to being loop mounted and plunged into a pool of liquid nitrogen. Diffraction data was collected at Diamond light source beamline I04, and processed using MOSFLM [23]. SeMet-substituted MAGE-A4 crystallized in 0.3 M Na-malonate pH 7, 20% PEG 3350, 10% ethylene glycol and 0.1M Bis-Tris propane pH 8.5, and crystals were cryo protected in a reservoir solution supplemented with 25% ethylene glycol. A two wavelength MAD data set was collected at the Swiss Light Source (SLS) on beamline X10SA at λ = 0.9782 Å (peak) and 0.97105 Å (remote). Data were integrated in MOSFLM[23], an SeMet positions located using SHELXD[24] and refined using program autoSHARP[25]. The initial model was built using BUCCANEER[26] and extended and completed manually using COOT[27]. The final refinement was performed using the 2.3 Å native dataset and the REFMAC[28] program to a final R_factor_ = 24.3%, R_free_ = 27.4%.

MAGE-A3 crystallized at 4°C in conditions containing 15% PEG 3350, 0.1M Mg Formate, and crystals were loop mounted and cryo protected in a reservoir solution supplemented with 25% ethylene glycol. Data were collected to 2.0Å resolution at Diamond light source beamline I24, and processed using XDS[29]. The structure was solved by molecular replacement using the program PHASER[30] and the structure of MAGE-A4 as a search model. Refinement was performed using PHENIX REFINE[31] to a final R_factor_ = 22.3%, R_free_ = 23.4%. A full summary of the data collection and refinement statistics are found in Table 1.

**Table 1:** Data collection and refinement statistics.

**Data collection statistics**			
	**Se Met MAGE-A4**	**MAGE-A4**	**MAGE-A3**
Space group	P 6_5_ 2 2	P 6_5_ 2 2	P 6_1_ 2 2
Cell dimensions, *a*,*b*,*c* (Å)	81.5, 81.5, 210.6	81.6, 81.6, 210.9	61.5, 61.5, 292.8
Angles α,β,γ (°)	90, 90, 120	90, 90, 120	90, 90, 120
Wavelength (Å)	0.978 (*pk*) 0.971 (*rm*)	0.979	0.9686
Resolution (Å)	35.2–2.97 (3.13–2.97)	42–2.30 (2.42–2.30)	39–2.07 (2.18–2.13)
R_merge_	0.15 (1.17)	0.07 (0.54)	0.06 (1.11)
R_p.i.m._	0.07 (0.71)	0.03 (0.24)	0.03 (0.36)
I/σI	10.2 (1.1)	13.9 (2.2)	19.9 (2.2)
Completeness (%)	95.4 (78.4)	99.7 (99.1)	100 (100)
Multiplicity	6.5 (3.2)	6.4 (5.0)	10.2 (10.8)
No. Unique reflections	8866 (1014)	19314 (2718)	21270 (1612)
**Refinement statistics**			
	**MAGE-A4**		**MAGE-A3**
Resolution	42–2.3		39–2.07
R_work_/R_free_ (%)	24.2 / 27.4		20.0 / 23.8
No. atoms			
Protein	1696		1663
Solvent	42		101
Ligand/ion	0		0
Average B factors (Å^2^)			
All atoms	47.5		53.7
Protein	47.6		53.7
Solvent	41.8		54.0
Wilson B	57.4		43.3
R.M.S. deviations			
Bond lengths (Å)	0.014		0.003
Bond angles (°)	1.41		0.761
Ramachandran plot			
Favoured (%)	92.7		98.6
Allowed (%)	100		100
PDBid	2WA0		4V0P

Values in parenthesis are for the highest resolution shell

### Small angle X-ray scattering

Small angle X-ray scattering measurements of MAGE-A3 in solution were performed at Diamond light source beamline B21 using a BIOSAXS robot for sample loading. Measurements were made using three different protein concentrations of 3.5, 1.75 and 0.88 mg/ml respectively in a buffer comprising 10 mM Hepes pH 7.5, 250 mM NaCl, 0.5 mM TCEP. The data were reduced and buffer contributions subtracted with the DawnDiamond software suite and analysed using the program SCATTER (www.bioisis.net). Pairwise distance distribution P(r) functions were calculated using GNOM[32] and compared to theoretical P(r) distributions from model coordinates in calculated in SCATTER. Real space scattering profiles of atomic models were calculated from atomic models using CRYSOL[33] and aligned and scaled to the experimental data using PRIMUS[34].

### Analytical ultracentrifugation

Sedimentation velocity AUC experiments were performed on a XL-I Analytical Ultracentrifuge using a Ti-50 rotor and cells with double-sector centrepieces (Beckman Coulter, Brea, CA, USA). Both MAGE-A3 and MAGE-A4 proteins were exhaustively dialysed against 10 mM HEPES (pH 7.5), 150 mM NaCl, and 0.5 mM TCEP and filtered through a 0.22 μm syringe filter prior to analysis. Samples were studied at 0.75 mg/ml (MAGE-A3) and 0.5 mg/ml (MAGE-A4) at 4°C, employing a rotor speed of 45,000 rpm. Absorbance data (280 nm) were analysed with SEDFIT[35] calculating c(s) distributions using partial specific volumes and buffer parameters calculated using the program SEDINTERP[36]. Theoretical sedimentation coefficients of model proteins were calculated from atomic models using the program HYDROPRO[37].

The structures reported here were deposited in the PDB, codes 2WA0 (MAGE-A4) and 4V0P (MAGE-A3).

## Results and Discussion

### Overall structure of the MAGE-A3 MHD domain

**Fig 1:**
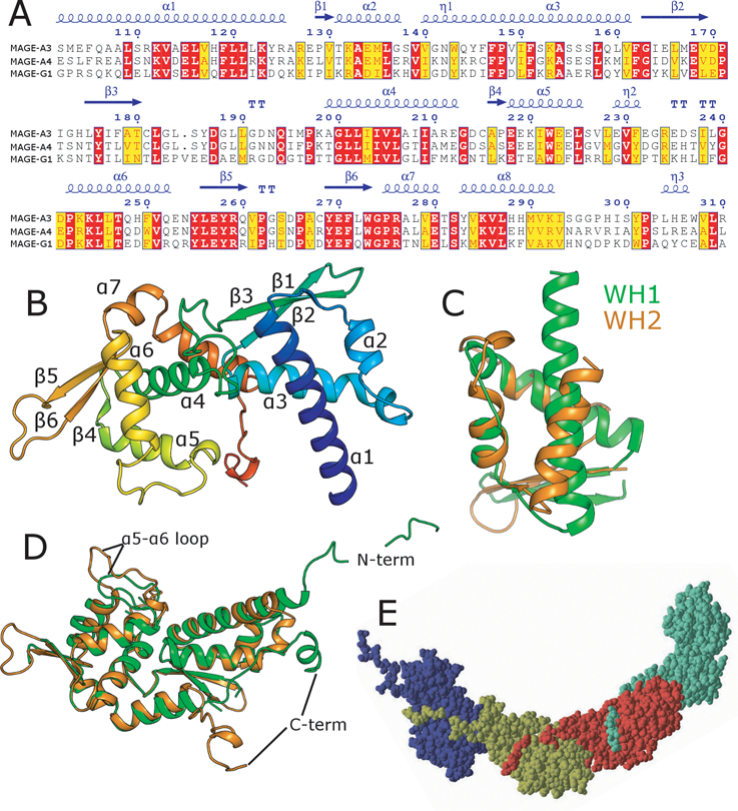
Structure of the MHD domain of MAGE-A3 and MAGE-A4. **(A)** Multiple sequence alignment of the MHD domains of MAGE-A3, MAGE-A4 and MAGE-G1, with the secondary structure elements of MAGE-A3 shown above for reference. **(B)** Overall structure of MAGE-A3 with secondary structure elements labelled. **(C)** Comparison of MAGE-A3 WH1 (green) and WH2 (orange). **(D)** Comparison of MAGE-A3 (orange) and MAGE-A4 (green). **(E)** Space filling representation of the arrangement of molecules in the MAGE-A4 crystals which form open ended interfaces via the N-terminal peptide sequence.

Crystals of the MAGE-A3 MHD domain were obtained using a construct spanning residues 104–314, and diffracted to 2.0 Å resolution at Diamond light source beamline I24. The structure was solved by molecular replacement using the experimentally phased structure of MAGE-A4 as a search model. The electron density map is of overall excellent quality with the entirety of the single molecule in the asymmetric unit being modelled with the exception of the final 4 residues at the C-terminus, which were not included in the final model due to disorder. The MHD domain structure consists of two highly modified winged helix WH domains WH1 and WH2 in a tandem arrangement linked by an extended β-hairpin (Fig 1A and 1B). Both WH domains feature the characteristic helix turn helix motif, which packs against a 3 stranded antiparallel β-sheet. Despite sharing no significant sequence homology, WH1 (residues 102–182) and WH2 (residues 199–276) can be superposed with an approximate 3.3 Å RMSD, with the biggest difference being the region connecting the second and third helices of the WH domain (often referred to as the helix turn helix HTH motif), which is of a compact helical character in WH1 but more extended in WH2 where an additional β-hairpin is formed (Fig 1B). The two WH domains are related by an approximate 160° rotation with a moderate interface between the two domains being formed by a combination of the connecting extended β-hairpin together with two additional α-helices from the C-terminus. The final 15 residues at the C-terminus form an extended peptide, with a short section of 3_10_ helix, which in the MAGE-A3 crystals forms an extensive interaction with a neighbouring molecule related by 2-fold crystallographic symmetry (Fig 1B).

### Overall structure of the MAGE-A4 MHD domain

Crystals of MAGE-A4 diffracted to 2.3 Å resolution at Diamond light source beamline I04, and the structure was solved by two wavelength MAD using SeMet derivatized protein. The electron density map is of overall high quality with the exception of three loop regions (spanning residues 171–175, 191–194 and 262–269) where the density was not of sufficient quality to build a reliable model. As would be expected given they share 64% sequence identities the MAGE-A4 structure is overall very similar to that of MAGE-A3 (1.48 Å RMSD over 209 aligned residues) with the relative positioning of the two domains being the same in both structures (Fig 1D). The most prominent difference between the two structures is the relative conformation of region between helices α5 and α6 (residues 230–241 on MAGE-A4) and the positioning of the extended N and C termini (Fig 1D). In MAGE-A4 the C-terminus is more closely associated with the rest of the molecule and forms a longer section of α-helix, which packs against the first and third helices of WH1. In contrast, the N-terminus of MAGE-A4, which includes 16 residues that were part of an uncleaved purification tag, is extended and forms an interface with neighbouring molecule related by crystal symmetry (Fig 1E). We note that it was not possible to obtain crystals of MAGE- A3 or MAGE-A4 when these extended C and N terminal sequences were removed, suggesting the interfaces are critical to the formation of the crystals.

### MAGE-A3 and MAGE-A4 peptide binding sites

**Fig 2:**
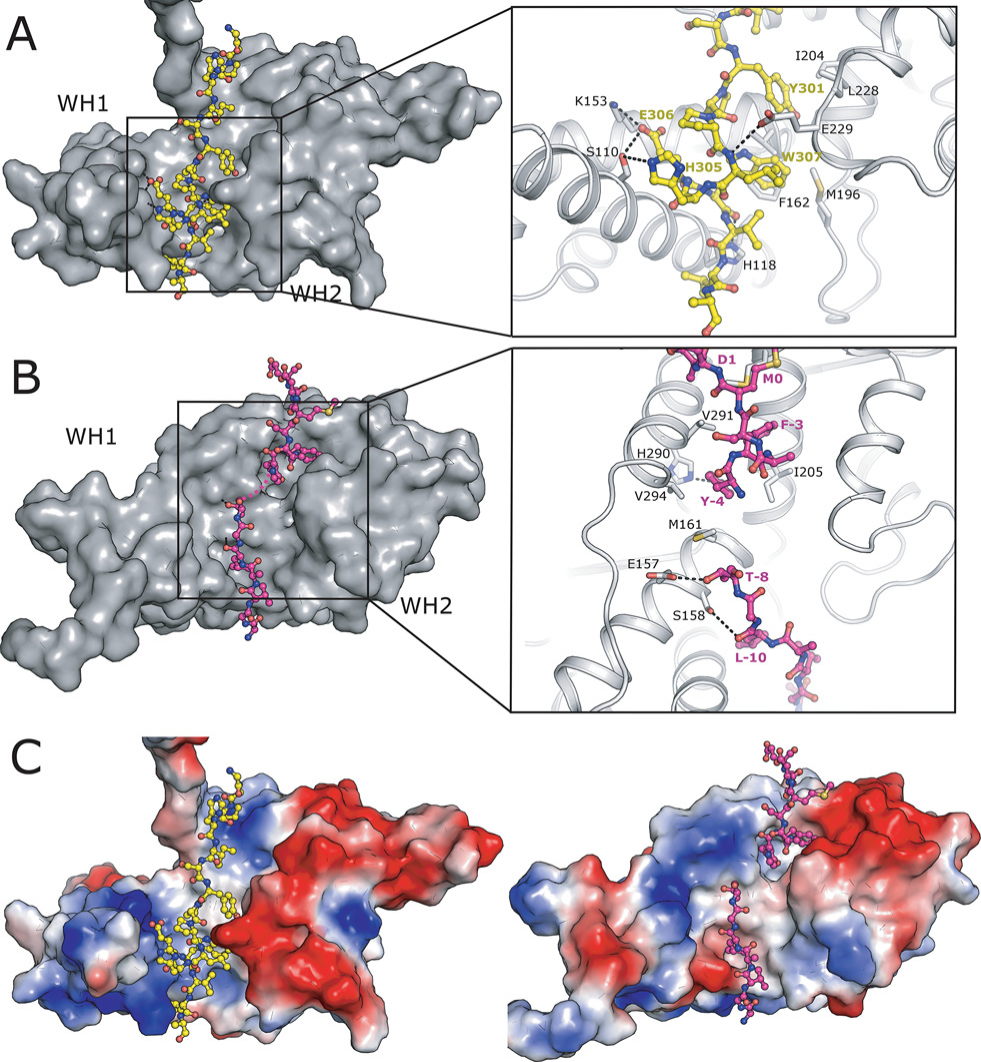
Interfaces of peptide sequences in the cleft between WH1 and WH2. **(A)** Surface representation of MAGE-A3 with the C-terminal peptide sequence shown in the ball and stick representation. The box shows a close up view with polar contacts shown as dashed lines and key interface residues labelled. **(B)** Surface representation of MAGE-A4 with the N-terminal peptide bound in the cleft, viewed from the same angle as in 2A. **(C)** Surface representation of MAGE-A3 (left) and MAGE-A4 (right) coloured according to electrostatic potential ± 5 kT/e. Both structures are viewed from the same angle as in 2A and 2B.

Given that the function of MAGE family proteins is likely to be in mediating protein-protein interactions in apoptosis, the finding of two separate instances of extended peptides binding to a conserved cleft between the two domains suggests a possible functional role for this region. Furthermore an analysis of this interface, and the nature of the contacts within it may provide an ideal starting point for the rational structure based design of targeted compounds to inhibit MAGE cellular function, such compounds may be useful in treating the various forms of cancer that are dependent on MAGE family proteins for their growth and survival. Despite the peptide sequences from MAGE-A3 and MAGE-A4 having different polarity, they both occupy an approximately equivalent position within a relatively deep groove formed between the two WH domains (Fig 2A). In MAGE-A3 the interface area is ~1650 Å^2^ per monomer, consists of a mixture of polar and non-polar residues, and includes 5 unique hydrogen bonds, 1 salt bridge and two prominent aromatic residues, Tyr 301 and Trp 307, that protrude deep in the groove and make extensive van der Waals interactions with hydrophobic residues (Fig 2A). In contrast to the situation in MAGE-A3, the electron density for the N-terminal, peptide sequence in MAGE-A4 is less well defined and the interpretation of the map in this region was more uncertain with only part of the peptide being modelled. Nevertheless, it is possible to identify three possible hydrogen bonds and two buried aromatic residue (Tyr -4 and Phe -3) (Fig 2B). Analysis of the electrostatic surfaces and sequence conservation patterns (Fig 2C) reveals that the chemical environment and residues forming the interface are quite different between the two proteins, indicating that compounds targeting these sites would have the potential to be relatively specific.

### Quaternary structure of MAGE-A3 and MAGE-A4

**Fig 3:**
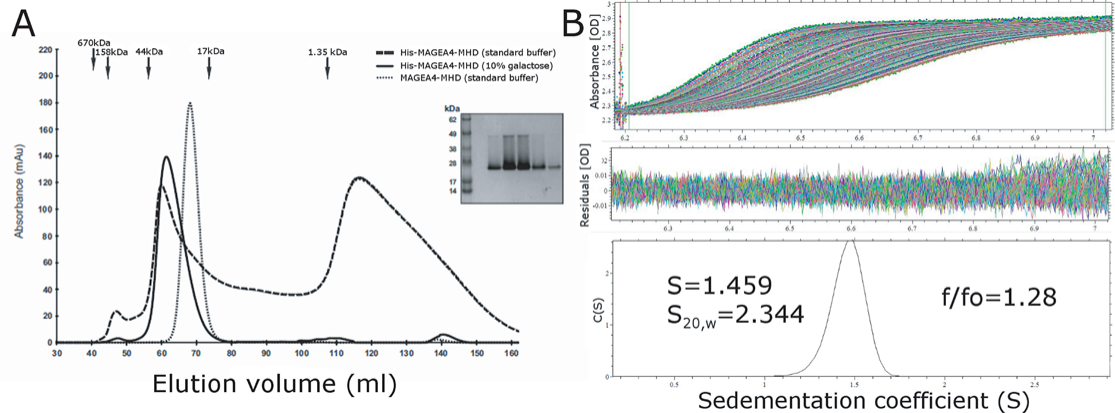
Analysis of MAGE-A4 in solution. **(A)** Gel filtration profiles of MAGE-A4 constructs with and without the N-terminal His tag, in the presence and absence of galactose. The elution volumes of size standards used for calibration are marked with black arrows. The inset shows a typical SDS PAGE gel of MAGE-A4 which shows two bands of approximately 25 and 45 kDa connected by a smear. **(B)** Analytical ultracentrifugation of cleaved MAGE-A4. The raw absorbance data plotted as a function of radius and time is shown in the top panel, the centre panel shows the distribution of residuals from the fit of the diffusion deconvoluted continuous distribution c(s) model, and the bottom panel shows the distribution of sedimentation coefficient values from the data fit.

Given the extensive nature of the interfaces created by the binging of the peptide sequences to this cleft we have investigated the possibility that they may have biological relevance and contribute towards oligomer formation in solution. In the case of MAGE-A4 this is unlikely since the interface is formed from residues in a purification tag, which are not part of the biological MAGE-A4 sequence. Analysis of MAGE-A4 on the PISA web service [38] indicates a monomer in solution with none of the other crystal contacts being probable dimer interfaces. Analysis of His tagged MAGE-A4 by size exclusion chromatography revealed an unusual behaviour with a significant amount of the protein eluting at volumes consistent with species much smaller than monomeric MAGE-A4, suggesting some kind of interaction with the column media. We were able to prevent this behaviour with addition of 10% galactose or by cleaving the tag, both of which resulted in peaks roughly consistent with monomeric species in solution (Fig 3A). Analysis of cleaved MAGE-A4 constructs by sedimentation velocity analytical ultracentrifugation reveals a single species with sedimentation coefficient S of 1.46 (S_w_^20^ of 2.34) (Fig 3B), consistent with a compact globular species with a molecular mass of 23360 ± 1937 Da, which matches very closely to the theoretical mass of MAGEA4, and the calculated hydrodynamic properties of the monomeric model (S_w_^20^ 2.43) using the program HYDROPRO.

**Fig 4:**
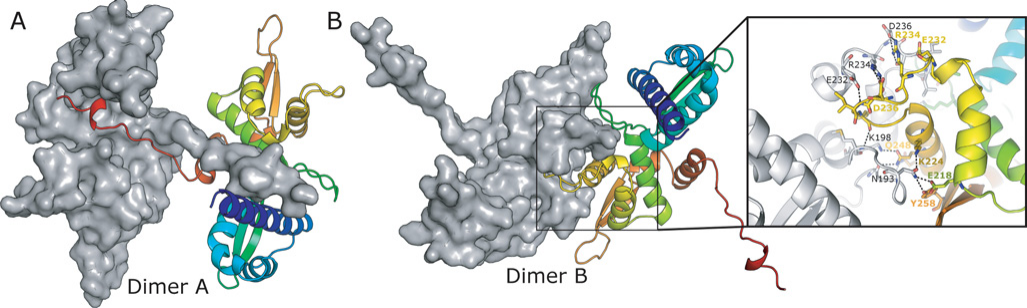
Structures of the two possible dimers present in the MAGE-A3 crystals. **(A)** Type A dimers linked by insertion of the C-terminal peptide into the cleft between WH1 and WH2. **(B)** Type B dimers linked by the extended β-hairpin, the insert shows a detailed view of the interface with interacting residues labelled and polar contacts shown as dashed lines.

In contrast to MAGEA4, PISA analysis of MAGE-A3 identified two potential biologically relevant dimer interfaces. In addition to the peptide interface detailed above (“dimer A”) a second interface is formed almost entirely by interactions from the second WH domain. This interface (“dimer B”) is only slightly less extensive (~1400 Å^2^ of interface area versus ~1650 Å^2^) and is formed by the extended β-hairpin connecting the two WH domains, together with the third helix on the second WH domain (α6) and the extended loop regions immediately preceding and following it (Fig 4A). This interface is predominantly polar in nature and contains 12 unique hydrogen bonds, and one unique salt bridge (Arg 234 to Asp 236) (Fig 4B).

**Fig 5:**
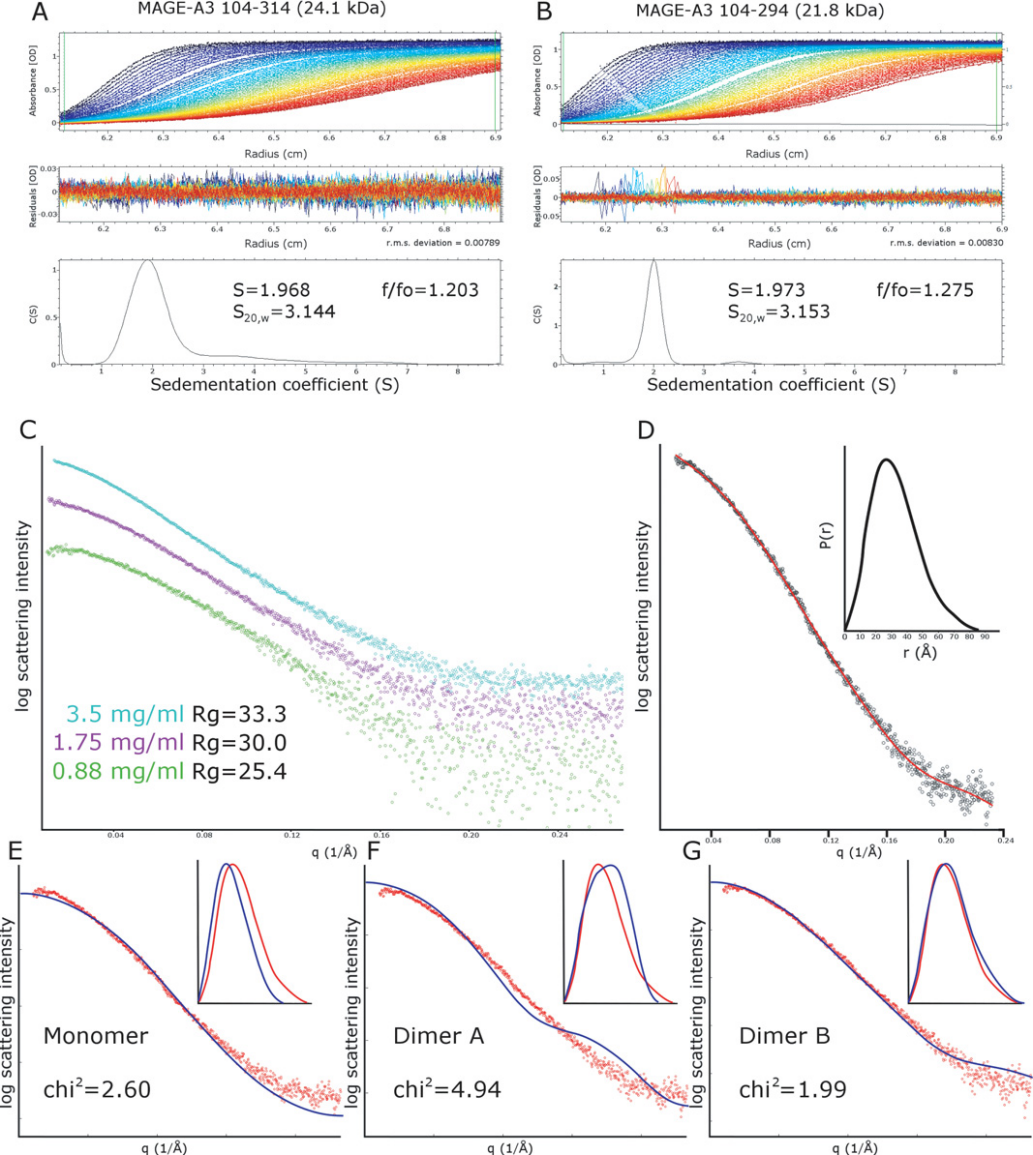
Analysis of MAGE-A3 in solution. (A-B) Analytical ultracentrifugation of MAGE-A3 constructs with (A) and without (B) the C-terminal peptide required to form type A dimers. The raw absorbance data plotted as a function of radius and time is shown in the top panel, the centre panel shows the distribution of residuals from the fit of the diffusion deconvoluted continuous distribution c(s) model, and the bottom panel shows the distribution of sedimentation coefficient values from the data fit. (C) Small angle X-ray scattering curves for MAGE-A3 (construct 104–314, containing the C-terminal peptide), collected at three different protein concentrations show significant concentration dependence in the Guinier region. (D) Distance distribution function P(r), calculated from the MAGE-A3 SAXS data, the main plot shows the fit of the P(r) function to the data with the distribution in the insert. (E-G) Comparisons of the experimental SAXS data and theoretical SAXS curves calculated from the MAGE-A3 monomer (E), dimer A (F) and dimer B (G). The main plot shows the fit in reciprocal space using the program CRYSOL[33], and the insert shows the fit in real space calculated with the program SCATTER (www.biosis.net).

In order to determine the oligomeric state of MAGE-A3 in solution and to distinguish between the two possible dimers, which may have similar hydrodynamic properties, we have produced a truncated construct MAGE-A3^104-294^ lacking the final 20 residues at the C-terminus of the crystallized construct. Analysis of either MAGE-A3 construct in solution by size exclusion chromatography reveals an unusual concentration dependant behaviour, with higher protein concentrations generally giving peaks consistent with greater apparent molecular masses (data not shown). Analysis of MAGE-A3 by sedimentation velocity analytical ultracentrifugation revealed that for both constructs a single compact globular species was present with sedimentation coefficient of S = 1.968 (S_20,w_ = 3.144) and S = 1.973 (S_20,w_ = 3.153) respectively, consistent in both cases with molecular masses of approximately 40 kDa (Fig 5A and 5B). These values are also in good agreement with the calculated theoretical sedimentation coefficients for the MAGE-A3 dimers, S_20w_ = 3.23 and 2.98 for dimer A and dimer B respectively, and although the experimental values fall somewhere in between the two calculated values the fact that a compact dimer is formed in the truncated construct strongly suggests that the dimerization in solution is formed via the dimer B interface, since there is no possibility to form the C-terminal dimer in this construct.

We have also performed an analysis of MAGE-A3 in solution by small angle X-ray scattering (SAXS). Data for the crystallized construct MAGE-A3^104-314^ collected at three different protein concentrations reveal a significant concentration dependence to the Gunier region of the scattering profile (Fig 5C), with the Gunier-based Rg decreasing by approximately 8 Å over a 4 fold dilution. To make best use of this data we have combined the scattering curves from the two lowest protein concentrations using the data at q>0.1 from the higher concentration sample to minimize noise at high q values (Fig 5D). This data can easily be transformed by indirect Fourier transform to a distance distribution function p(r), with the reciprocal and real space radius of gyration values broadly in agreement (Rg = 25.4 and 26.5 respectively). Comparing this data to the calculated data obtained using the monomeric MAGE-A3 (Fig 5E), dimer A (Fig 5F) and dimer B (Fig 5G) models reveals a significantly better fit for dimer B than either dimer A or monomer, when comparing either in reciprocal or real space (chi^2^ of 1.99 versus 4.94 or 2.6 respectively).

**Fig 6:**
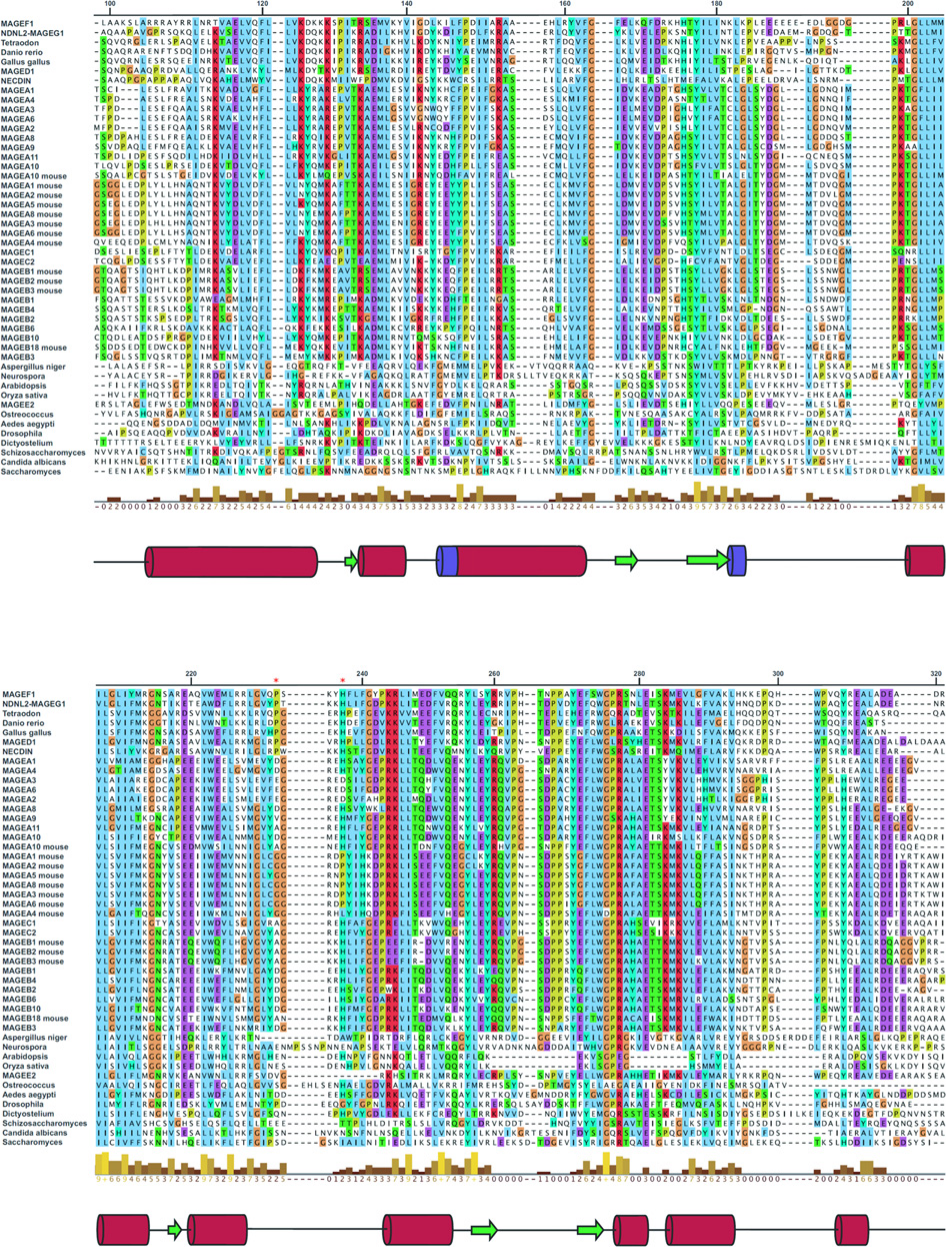
Sequence alignment of the MAGE homology domain of representative human MAGE sequences. Sequences were aligned using CLUSTALX and visualised with Jalview. Alignment numbering is according to MAGE-A4 residue number. Secondary structure is aligned to the MAGE-A4 structure, with α-helices as red cylinders, β-strands as green arrows and 310 helices as purple cylinders. Residue conservation is represented by the histogram, with a scale from 0 (no conservation) to 9, with complete identity denoted by an asterisk. Residues E232 and D236 in the dimer interface of MAGEA3 (marked with *) are found only in MAGEA3, MAGEA6 and MAGEA2; the corresponding residues in MAGEA3 are D233 and H237.

Taken together it is clear that MAGE-A3 is predominantly a dimer in solution and that dimer corresponds to dimer B, although it is possible that at higher concentrations these dimeric units may further associate via the C-terminal peptide to form larger open ended oligomers. A comparison of the MAGE-A3 and MAGE-A4 structures in the dimerization interface reveals a significantly different conformation of the loop between the α5 and α6, which is extensively involved in the dimer interface in MAGE-A3 but in MAGE-A4 adopts conformation which would make significant steric clashes with its symmetry counterpart, explaining why the same interface was not formed in MAGE-A4. Looking at the sequence in this region two significant substitutions are present, E232D and D236H, which would likely cause the loss of four hydrogen bonds and two salt bridges from the interface. A phylogenetic analysis of the entire human MAGE family (Fig 6), reveals these residues are present only in the closest two MAGE-A3 homologues, MAGE-A6 and MAGE-A2, indicating that the interface has arisen at the relatively late stages of MAGE family evolution. Another feature common to MAGE-A4 and MAGE-A3 is an additional 100 amino acids N-terminal to the MHD domains, which are thought to be predominantly unstructured and may contribute towards the oligomeric sate of the full length protein *in vivo*.

### Comparisons of MAGE-A3 and MAGE-A4 with MAGE-G1

**Fig 7:**
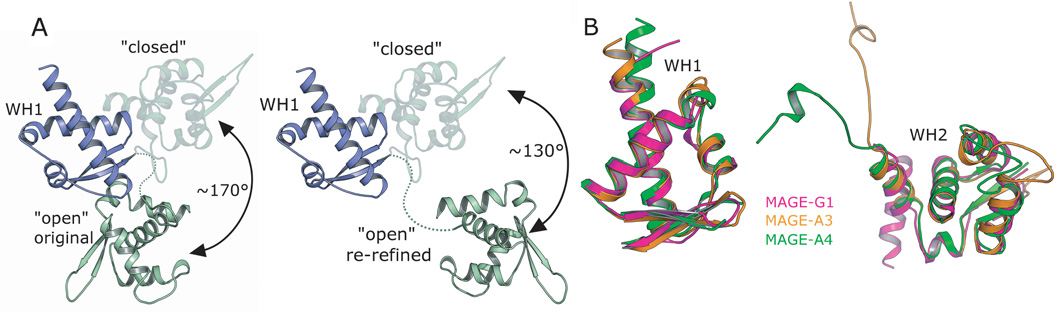
Comparisons between MAGE-G1 and MAGE-A3/A4. **(A)** Comparison of the relative conformations of WH1 and WH2 in the MAGE-G1 structure (open form) and in the MAGE-A3/A4 structures (closed form, shown as semi-transparent cartoons), the original connectivity is shown on the left and the re-refined on the right hand side. **(B)** Superposition of the MAGE-A3 (orange), MAGE-A4 (green) and MAGE-G1 (pink) structures on the basis of the individual WH1 and WH2 domains.

The only other MAGE domain structure in the PDB to date is the structure of the MAGE-G1 NSE-1 complex [15]. Comparing this structure to both MAGE-A3 and MAGE-A4 reveals in both cases that there are significant differences in the relative positioning of the WH1 and WH2 domains, which in MAGE-G1 are more distantly separated, and do not share any significant interface. Using WH1 as a guide, a domain rotation of approximately 170° and translation of ~30Å would be required to put WH2 in the position adopted by MAGE-G1 (Fig 7A) with the hinge region being part of the linker connecting WH1 and WH2. Taken individually, the WH domains of MAGE-A3 and MAGE-A4 can be aligned to MAGE-G1 with R.M.S.D. values of 1.3 Å and 1.0 Å for WH1, and 1.5 Å and 1.7 Å for WH2 respectively. The most prominent difference lies in the loop regions of WH2, several of which are disordered in the MAGE-G1 structure (Fig 7B).

Given the fact that MAGE-G1 and MAGE-A3/4 share such a highly conserved core structure, it is interesting to speculate whether these structures are representative of two different conformational states, “Open” and “Closed” forms respectively. An examination of the regions of WH1 on MAGE-G1 that contribute towards the NSE-1 interface reveals that they are not fully accessible in the “Closed” form structures, suggesting that a conformational change is required for MAGE–RING domain binding and that targeting the peptide binding groves with compounds which bind to and stabilize preferentially the “Closed” conformation may be a valid therapeutic route for cancer treatment.

### An alternative model for the MAGE-G1 NSE-1 interface

**Fig 8:**
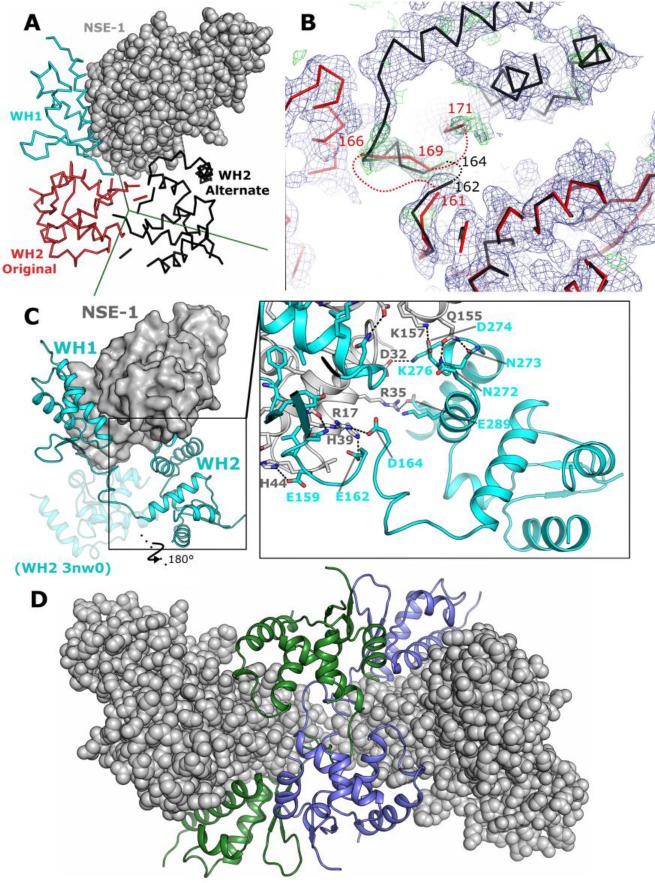
Re-analysis of the MAGE-G1 NSE1 model. **(A)** View of the original (red ribbon) and alternate (black ribbon) choices around the crystallographic symmetry axis (shown as green lines). A single NSE-1 is shown as grey spheres. **(B)** Electron density maps in the region connecting WH1 and WH2. The 2F_o_-1F_c_ (blue) and F_o_-F_c_ (green) electron density maps (calculated with all atoms between 161 and 171 omitted from the model) are shown contoured at 0.9 σ and 2.4 σ respectively with the domains coloured as for panel A. **(C)** Comparison of the interfaces between the original (semi-transparent cartoon) and alternative (opaque cartoon) MAGE-G1 models and NSE-1 (shown in the surface representation). The insert shows a detailed view of the additional interface in the alternate model with interacting residues labelled and shown in the stick format and polar contacts shown as dashed lines. **(D)** Possible MAGE-G1 NSE-1 hetero-tetramer found in the MAGE-G1 NSE-1 crystallographic asymmetric unit with the two MAGE-G1 monomers (shown in green and blue) topologically interlinked.

**Fig 9:**
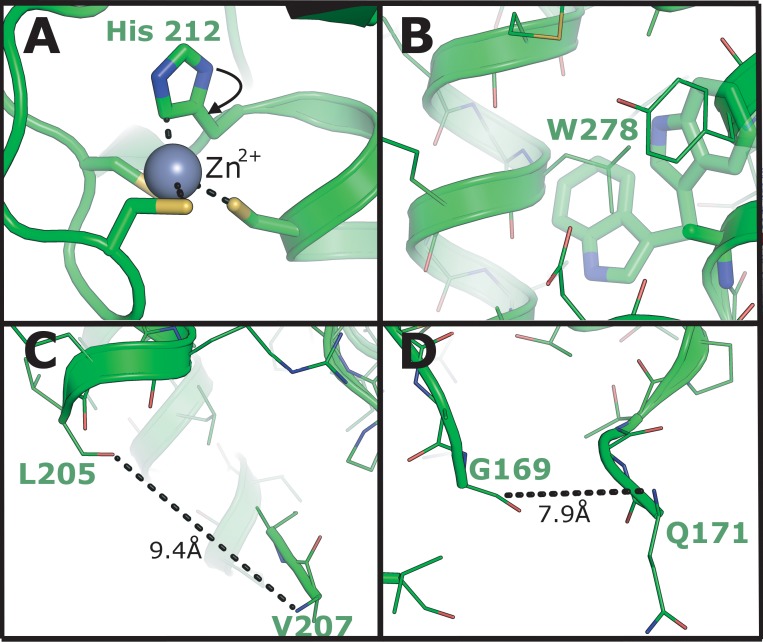
Examination of physically unlikely features in the MAGE-G1 NSE-1 complex structure 3NW0. (A) Zinc ion coordination in the NSE-1 structure by three cysteine residues and a histidine which clearly needs to be rotated to make chemical sense. (B) Tryptophan sidechain which is truncated at Cβ in the 3NW0 model, with insufficient space for any of the possible rotamers (shown in a semi-transparent stick representation) to be accommodated without significant steric clashes. (C-D) Large gaps in the structure with distances incompatible with a single missing residue. Gaps are shown by dashed lines with distances and residue registers labelled accordingly.

We have noted while examining the MAGE-G1 NSE-1 complex structure that the linker region connecting WH1 and WH2 was absent from the model and the electron density in this region was poor. As the missing residues lie close to a crystallographic 2-fold symmetry axis, the termini of the various domains are positioned such that there are two alternative ways to connect them (Fig 8A). To investigate which of these two possibilities is correct, and in the absence of other experimental information, we have downloaded the structure factors and performed a re-refinement of the structure, testing if the data support the alternative connectivity. Our re-refined model has slightly improved refinement and geometry statistics, and we were able to correct a number of minor model building errors in the original coordinates (Fig 9, Table 2). Electron density maps covering the connecting region, (calculated omitting contributions from residues in the linker region) show significant positive density features generally supporting the alternative connection (Fig 8B), although we were unable to completely connect the two domains in this way with 2 residues (163 and 164) being absent from the re-refined model.

**Table 2:** Refinement statistics for the original and re-refined MAGE-G1 NSE-1 structure.

	Original	Re-refined
Resolution	40–2.75 #	32.3–2.92 #
R_work_/R_free_ (%)	21.9/27.1	23.7/26.8
No. atoms		
Protein	3516*	3532
Solvent	0 *	0
Ligand/ion	3 *	2
Average B factors (Å^2^)		
Protein	110	100
Ligand/ion	107	74
Wilson B	111	90
R.M.S. deviations		
Bond lengths (Å)	0.011	0.003
Bond angles (°)	1.523	0.69
Ramachandran plot		
Favoured (%)	87.8	94.05
Allowed (%)	96.41	100
PDBid	3NW0	?

# Reported resolution and statistics for the original MAGE-G1 NSE-1 data do not match the structure factors uploaded to wwPDB entry 3NW0.

* Reported model contents do not match wwPDB entry 3NW0 (PDB entry contains 3476 protein atoms, 2 Zn^2+^ ions, 2 Mg^2+^ ions and 17 water molecules.

Importantly for the biological interpretation of the interaction between MAGE-G1 and NSE-1 (and MAGE RING interfaces in general), the re-refined interface between NSE-1 and MAGE-G1 contains contributions from both the N and C-terminal WH domains. In the new interface the N-terminal WH domain of NSE-1 is sandwiched between the two WH domains of MAGE-G1 with additional contacts coming from residues in the linker region and the C-terminal WH domain WHB, some of which involve residues in the second WH domain of NSE-1. Analysis of the interface by the PISA software identified an additional 385 Å^2^ of interface area (1385 Å^2^ total), 11 additional hydrogen bonds and 7 additional salt bridges (Fig 8C). Support for this interpretation comes from the fact that the *in vitro* protein-protein interaction data found that both the N and C terminal WH domains of MAGE-G1 were required for NSE-1 binding [15]. On the other hand if the two WH domains are connected in this way, the symmetry at this position would mean that two of the MAGE-G1 NSE-1 complexes are almost certainly topologically interlinked in the crystals. Whilst this type of arrangement is not totally unprecedented, it is difficult to imagine how the crystals were able to form and grow. One possible explanation is that the MAGE-G1 NSE-1 complex exists as a higher order oligomeric species in solution, possibly a heterotetramer (Fig 8D), which has an ordered pathway for addition of monomers. Alternatively it is a possibility that MAGE-G1 has been subjected to proteolytic cleavage prior to or during the crystallization experiment. In this case the complexes are not in fact interlinked and the lack of complete connecting density could be explained by the cleavage event. Serendipitous proteolysis is not uncommon in crystallization and we are aware of the power of the constraints of crystallization in selecting specifically for infrequent events. In any case it would appear that additional experimentation may be required to resolve this question.

## Conclusions

In this study we present the first and third MAGE domain structures to be determined. The two structures both show a tandem arrangement of modified WH domains, which form the same relative positioning of the two domains and an overall high degree of structural similarity (RMSD 1.5 Å). Surprisingly both proteins contained an extended peptide sequence bound tightly to a groove formed between the two domains suggesting a possible biological role for this region in protein-protein interactions.

The oligomeric state of the various MAGE proteins may also play a role in their function as protein interaction modules *in vivo*. We have found MAGE-A4 to be a monomer in solution whereas MAGE-A3 is a dimer, linked by an extensive interface that predominantly contains residues from WH2. The majority of the equivalent residues to those involved in the MAGE-G1 NSE-1 interface, are not part of the MAGE-A3 dimer, suggesting that binding and dimerization need not be mutually exclusive.

We have also performed a re-examination of the crystal structure data on the MAGE-G1 NSE-1 complex and suggest an alternative way in which the N and C terminal WH domains of MAGE-G1 can be connected. Importantly this configuration gives a significantly more extensive interface for NSE-1 binding and is consistent with experimental data showing both WH domains were required for full NSE-1 binding activity. A comparison of these various structures reveals that significant domain movements would be required to place the domains in the same relative positions (regardless of whether the original or alternate arrangement of MAGE-G1 is chosen for comparison).

One key aspect of MAGE domain function is the question of whether these domain rearrangements represent different functional states of the MAGE protein biological function. Several of the regions that contribute to the MAGE-G1 NSE-1 interface are no longer solvent accessible in the conformation adopted by free MAGE-A3 or MAGE-A4. This would suggest that the “closed” conformations adopted by MAGE-A3 and MAGE-A4 to which peptide sequences are bound would be incapable of forming, or require substantial rearrangements to form associations with RING family members. This “conformational plasticity” between the free and RING protein-bound states suggests selective and specific pharmaceutical strategies that target conformation rather than enzymatic activity. Thus the peptide binding sites of MAGE-A3 and MAGE-A4 may be important for MAGE regulation *in vivo*, and drug molecules targeting these sites could potentially lock the MHD in the “closed” conformation, prevent binding to their RING domain partners, and antagonize their proliferative and survival activities in cancer cells.

## Supporting Information

S1 File Validation report of MAGEA4 structure PDB:2WA0. (PDF)

S2 File Validation report of MAGEA4 structure PDB:4V0P. (PDF)
