# Supplementary material for: Structures of Two Melanoma-Associated Antigens Suggest Allosteric Regulation of Effector Binding
Source: PLoS One. 2016 Feb 24;11(2):e0148762. doi: 10.1371/journal.pone.0148762 (PMC4766014; doi:10.1371/journal.pone.0148762)
Supplement: S2 File — (PDF) [file pone.0148762.s002.pdf]

Full wwPDB X-ray Structure Validation Report (i)

Sep 26, 2014 – 11:28 AM EDT

PDB ID	:	4V0P
Title	:	Crystal structure of the MAGE homology domain of human MAGE-A3
Authors	:	Newman, J.A.; Aitkenhead, H.; Cooper, C.D.O.; Pinkas, D.M.; von Delft, F.; Arrowsmith, C.H.; Edwards, A.; Bountra, C.; Gileadi, O.
Deposited on	:	2014-09-17
Resolution	:	2.07 Å(reported)

This is a full wwPDB validation report for a publicly released PDB entry.
We welcome your comments at validation@mail.wwpdb.org
A user guide is available at http://wwpdb.org/ValidationPDFNotes.html

The following versions of software and data (see references) were used in the production of this report:

MolProbity	:	4.02b-467
Mogul	:	1.16 November 2013
Xtriage (Phenix)	:	dev-1439
EDS	:	trunk23956
Percentile statistics	:	23426
Refmac	:	5.8.0049
CCP4	:	6.3.0 (Settle)
Ideal geometry (proteins)	:	Engh & Huber (2001)
Ideal geometry (DNA, RNA)	:	Parkinson et. al. (1996)
Validation Pipeline (wwPDB-VP)	:	trunk23956

1 Overall quality at a glance (i)

The reported resolution of this entry is 2.07 Å.

Percentile scores (ranging between 0-100) for global validation metrics of the entry are shown in the following graphic. The table shows the number of entries on which the scores are based.

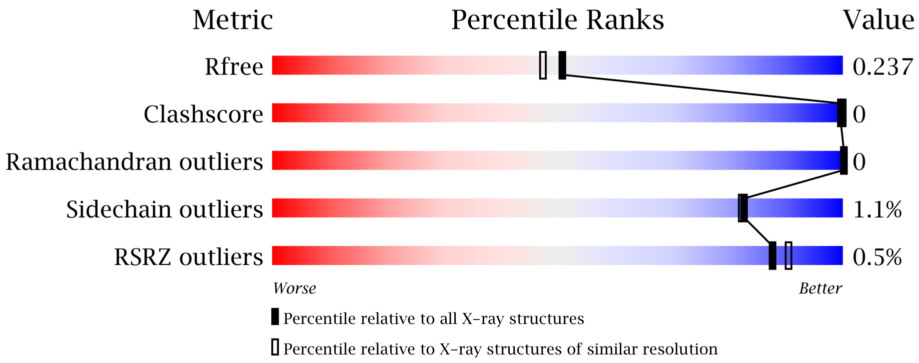

Metric	Whole archive (#Entries)	Similar resolution (#Entries, resolution range(Å))
R_{free}	77520	1463 (2.08-2.04)
Clashscore	88313	1571 (2.08-2.04)
Ramachandran outliers	86584	1561 (2.08-2.04)
Cα geometry	86677	1559 (2.08-2.04)
Sidechain outliers	86556	1561 (2.08-2.04)
RSRZ outliers	77580	1465 (2.08-2.04)

The table below summarises the geometric issues observed across the polymeric chains and their fit to the electron density. The red, orange, yellow and green segments on the lower bar indicate the fraction of residues that contain outliers for >=3, 2, 1 and 0 types of geometric quality criteria. The upper red bar (where present) indicates the fraction of residues that have poor fit to the electron density.

Mol	Chain	Length	Quality of chain
1	A	213	

2 Entry composition (i)

There are 2 unique types of molecules in this entry. The entry contains 1764 atoms, of which 0 are hydrogen and 0 are deuterium.

In the tables below, the ZeroOcc column contains the number of atoms modelled with zero occupancy, the AltConf column contains the number of residues with at least one atom in alternate conformation and the Trace column contains the number of residues modelled with at most 2 atoms.

- Molecule 1 is a protein called MELANOMA-ASSOCIATED ANTIGEN 3.

Mol	Chain	Residues	Atoms					ZeroOcc	AltConf	Trace
			Total	C	N	O	S			
1	A	209	1663	1089	268	298	8	0	0	0

There are 2 discrepancies between the modelled and reference sequences:

Chain	Residue	Modelled	Actual	Comment	Reference
A	102	SER	-	EXPRESSION TAG	UNP P43357
A	103	MET	-	EXPRESSION TAG	UNP P43357

- Molecule 2 is water.

Mol	Chain	Residues	Atoms		ZeroOcc	AltConf
			Total	O		
2	A	101	101	101	0	0

3 Residue-property plots (i)

These plots are drawn for all protein, RNA and DNA chains in the entry. The first graphic for a chain summarises the proportions of errors displayed in the second graphic. The second graphic shows the sequence view annotated by issues in geometry and electron density. Residues are color-coded according to the number of geometric quality criteria for which they contain at least one outlier: green = 0, yellow = 1, orange = 2 and red = 3 or more. A red dot above a residue indicates a poor fit to the electron density (RSRZ > 2). Stretches of 2 or more consecutive residues without any outlier are shown as a green connector. Residues present in the sample, but not in the model, are shown in grey.

- Molecule 1: MELANOMA-ASSOCIATED ANTIGEN 3

Chain A:

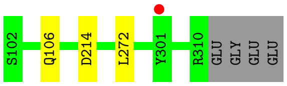

4 Data and refinement statistics ⓘ

Property	Value	Source
Space group	P 61 2 2	Depositor
Cell constants<br>a, b, c, α, β, γ	61.47Å 61.47Å 292.83Å<br>90.00° 90.00° 120.00°	Depositor
Resolution (Å)	39.39 – 2.07<br>53.24 – 2.07	Depositor<br>EDS
% Data completeness<br>(in resolution range)	99.9 (39.39-2.07)<br>99.9 (53.24-2.07)	Depositor<br>EDS
R_{merge}	0.06	Depositor
R_{sym}	(Not available)	Depositor
$< I/\sigma(I) >$ [1]	1.96 (at 2.07Å)	Xtriage
Refinement program	PHENIX (PHENIX.REFINE)	Depositor
R, R_{free}	0.200 , 0.238<br>0.202 , 0.237	Depositor<br>DCC
R_{free} test set	1013 reflections (4.79%)	DCC
Wilson B-factor (Å^2)	43.2	Xtriage
Anisotropy	0.021	Xtriage
Bulk solvent k_{sol}(e/Å^3), B_{sol}(Å^2)	0.32 , 40.5	EDS
Estimated twinning fraction	No twinning to report.	Xtriage
L-test for twinning[2]	$< \|L\| > = 0.47$, $< L^2 > = 0.30$	Xtriage
Outliers	0 of 21158 reflections	Xtriage
F_o,F_c correlation	0.95	EDS
Total number of atoms	1764	wwPDB-VP
Average B, all atoms (Å^2)	53.0	wwPDB-VP

Xtriage's analysis on translational NCS is as follows: *The largest off-origin peak in the Patterson function is 7.02% of the height of the origin peak. No significant pseudotranslation is detected.*

[1]Intensities estimated from amplitudes.

[2]Theoretical values of $< |L| >$, $< L^2 >$ for acentric reflections are 0.5, 0.375 respectively for untwinned datasets, and 0.333, 0.2 for perfectly twinned datasets.

5 Model quality (i)

5.1 Standard geometry (i)

The Z score for a bond length (or angle) is the number of standard deviations the observed value is removed from the expected value. A bond length (or angle) with $|Z| > 5$ is considered an outlier worth inspection. RMSZ is the root-mean-square of all Z scores of the bond lengths (or angles).

Mol	Chain	Bond lengths		Bond angles	
		RMSZ	$\#\|Z\| > 5$	RMSZ	$\#\|Z\| > 5$
1	A	0.24	0/1708	0.41	0/2320

There are no bond length outliers.

There are no bond angle outliers.

There are no chirality outliers.

There are no planarity outliers.

5.2 Close contacts (i)

In the following table, the Non-H and H(model) columns list the number of non-hydrogen atoms and hydrogen atoms in the chain respectively. The H(added) column lists the number of hydrogens added by MolProbity. The Clashes column lists the number of clashes within the asymmetric unit, and the number in parentheses is this value normalized per 1000 atoms of the molecule in the chain. The Symm-Clashes column gives symmetry related clashes, in the same way as for the Clashes column.

Mol	Chain	Non-H	H(model)	H(added)	Clashes	Symm-Clashes
1	A	1663	0	1663	1	0
2	A	101	0	0	1	0
All	All	1764	0	1663	1	0

Clashscore is defined as the number of clashes calculated for the entry per 1000 atoms (including hydrogens) of the entry. The overall clashscore for this entry is 0.

All (1) close contacts within the same asymmetric unit are listed below.

Atom-1	Atom-2	Distance(Å)	Clash(Å)
1:A:106:GLN:NE2	2:A:2001:HOH:O	2.47	0.47

There are no symmetry-related clashes.

5.3 Torsion angles

5.3.1 Protein backbone (i)

In the following table, the Percentiles column shows the percent Ramachandran outliers of the chain as a percentile score with respect to all X-ray entries followed by that with respect to entries of similar resolution.

The Analysed column shows the number of residues for which the backbone conformation was analysed, and the total number of residues.

Mol	Chain	Analysed	Favoured	Allowed	Outliers	Percentiles	
1	A	207/213 (97%)	204 (99%)	3 (1%)	0	100	100

There are no Ramachandran outliers to report.

5.3.2 Protein sidechains (i)

In the following table, the Percentiles column shows the percent sidechain outliers of the chain as a percentile score with respect to all X-ray entries followed by that with respect to entries of similar resolution. The Analysed column shows the number of residues for which the sidechain conformation was analysed, and the total number of residues.

Mol	Chain	Analysed	Rotameric	Outliers	Percentiles	
1	A	180/184 (98%)	178 (99%)	2 (1%)	83	82

All (2) residues with a non-rotameric sidechain are listed below:

Mol	Chain	Res	Type
1	A	214	ASP
1	A	272	LEU

Some sidechains can be flipped to improve hydrogen bonding and reduce clashes. All (2) such sidechains are listed below:

Mol	Chain	Res	Type
1	A	118	HIS
1	A	249	HIS

5.3.3 RNA (i)

There are no RNA chains in this entry.

5.4 Non-standard residues in protein, DNA, RNA chains (i)

There are no non-standard protein/DNA/RNA residues in this entry.

5.5 Carbohydrates (i)

There are no carbohydrates in this entry.

5.6 Ligand geometry (i)

There are no ligands in this entry.

5.7 Other polymers (i)

There are no such residues in this entry.

5.8 Polymer linkage issues

There are no chain breaks in this entry.

6 Fit of model and data (i)

6.1 Protein, DNA and RNA chains (i)

In the following table, the column labelled '#RSRZ> 2' contains the number (and percentage) of RSRZ outliers, followed by percent RSRZ outliers for the chain as percentile scores relative to all X-ray entries and entries of similar resolution. The OWAB column contains the minimum, median, 95th percentile and maximum values of the occupancy-weighted average B-factor per residue. The column labelled 'Q< 0.9' lists the number of (and percentage) of residues with an average occupancy less than 0.9.

Mol	Chain	Analysed	<RSRZ>	#RSRZ>2			OWAB(Å^2)	Q<0.9
1	A	209/213 (98%)	0.12	1 (0%)	88	90	34, 50, 78, 99	0

All (1) RSRZ outliers are listed below:

Mol	Chain	Res	Type	RSRZ
1	A	301	TYR	2.1

6.2 Non-standard residues in protein, DNA, RNA chains (i)

There are no non-standard protein/DNA/RNA residues in this entry.

6.3 Carbohydrates (i)

There are no carbohydrates in this entry.

6.4 Ligands (i)

There are no ligands in this entry.

6.5 Other polymers (i)

There are no such residues in this entry.
